# Understanding the Cathode‐Electrolyte Interfacial Chemistry in Rechargeable Magnesium Batteries

**DOI:** 10.1002/advs.202401536

**Published:** 2024-04-06

**Authors:** Hucheng Shi, Guixin Wang, Zhechen Wang, Lin Yang, Shu Zhang, Shanmu Dong, Baihua Qu, Aobing Du, Zhenyou Li, Xiaoyuan Zhou, Guanglei Cui

**Affiliations:** ^1^ Qingdao Institute of Bioenergy and Bioprocess Technology Chinese Academy of Sciences Qingdao 266101 China; ^2^ Center of Materials Science and Optoelectronics Engineering University of Chinese Academy of Sciences Beijing 100049 China; ^3^ Shandong Energy Institute Qingdao 266101 China; ^4^ Qingdao New Energy Shandong Laboratory Qingdao 266101 China; ^5^ College of Materials Science and Engineering National Engineering Research Center for Magnesium Alloys Chongqing University Chongqing 400044 China

**Keywords:** cathode‐electrolyte interphase, interfacial chemistry, rechargeable magnesium batteries, solvation structure

## Abstract

Rechargeable magnesium batteries (RMBs) have garnered significant attention due to their potential to provide high energy density, utilize earth‐abundant raw materials, and employ metal anode safely. Currently, the lack of applicable cathode materials has become one of the bottleneck issues for fully exploiting the technological advantages of RMBs. Recent studies on Mg cathodes reveal divergent storage performance depending on the electrolyte formulation, posing interfacial issues as a previously overlooked challenge. This minireview begins with an introduction of representative cathode‐electrolyte interfacial phenomena in RMBs, elaborating on the unique solvation behavior of Mg^2+^, which lays the foundation for interfacial chemistries. It is followed by presenting recently developed strategies targeting the promotion of Mg^2+^ desolvation in the electrolyte and alternative cointercalation approaches to circumvent the desolvation step. In addition, efforts to enhance the cathode‐electrolyte compatibility via electrolyte development and interfacial engineering are highlighted. Based on the abovementioned discussions, this minireview finally puts forward perspectives and challenges on the establishment of a stable interface and fast interfacial chemistry for RMBs.

## Graphical abstract

Interface chemistry has always been a key issue for various batteries owing to its significant effect on electrochemical performance. This minireview offers a thorough summary of cathode‐electrolyte interfacial chemistry in rechargeable magnesium batteries, including the representative interfacial phenomena, the detailed desolvation process in various electrolytes, the compatibility between cathode and electrolytes, and the perspectives on favorable interphase construction.

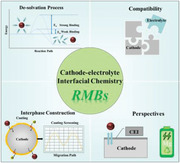

## 1 Introduction

Electrochemical energy storage devices are of great significance for the sustainable development of human production and life.^[^ ^1^ ^]^ Li‐ion batteries (LIBs), the most outstanding battery technology with superior performance, have revolutionized our daily lives through their wide application in portable electronic products, electric vehicles, and large‐scale energy storage sectors.^[^ ^2^, ^3^ ^]^ However, the limited lithium resources and uneven distribution of the minerals have raised concerns about the stability of the supply chain of current LIBs technology.^[^ ^4^, ^5^ ^]^ Meanwhile, the rapidly growing demands have stimulated the development of post‐lithium battery systems, such as Na‐ion batteries, Zn‐ion batteries, Al‐ion batteries, and Mg‐ion batteries.^[^ ^6^ ^]^ As one of the most promising alternatives, rechargeable Mg batteries (RMBs) have received increasing attention because of abundant Mg resources and high safety.^[^ ^7^ ^]^ Compared to Li, the Mg metal anode is less susceptible to forming detrimental metal dendrites during electrochemical plating, meanwhile providing a higher volumetric capacity (3833 mAh cm^−3^).^[^ ^6^ ^]^ Nevertheless, high‐performance Mg batteries must be built based on the development of viable intercalation chemistry of Mg^2+^, and the lack of appropriate cathode materials with efficient reversible Mg^2+^ storage properties is currently a key issue in the development of RMBs.^[^ ^8^ ^]^

**Figure 1:**
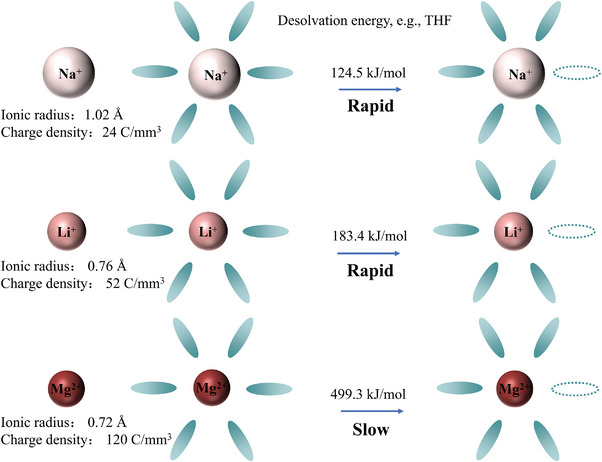
Comparison of ionic radii, charge densities, and de‐solvation energies of Na^+^, Li^+^, and Mg^2+^. According to the literature,^[^ ^10^ ^]^ the desolvation energies were calculated for tetrahydrofuran(THF)‐solvated cations.

So far, extensive efforts have been devoted to understanding and enhancing the solid‐state diffusion process of Mg^2+^ within the lattice of intercalation‐type cathodes. As is known, divalent Mg^2+^ with a significant polarizing effect can generate a strong interaction with the polarized anions of the cathode host, resulting in slow Mg^2+^ diffusion kinetics and poor Mg^2+^‐storage performance.^[^ ^9^ ^]^ The strong polarization properties of Mg^2+^ also induce stronger interactions with polarized species or moieties of solvent molecules in the electrolyte, leading to a more rigid solvation structure of Mg^2+^. Therefore, the solvated Mg^2+^ at the cathode electrolyte interface must overcome a higher desolvation energy barrier to remove the surrounding solvent and, consequently, enter the lattice framework of the cathodes. In general, the de‐solvation energy for Mg^2+^ is as high as more than double the value of Li^+^ or Na^+^ in different organic solvents,^[^ ^10^ ^]^ as shown in **Figure** **1**. According to DFT calculations, the energy barrier for breaking the Mg─Cl bonds in common Cl‐containing Mg‐based electrolytes can be twice that for Mg^2+^‐diffusion within bulk TiS_2_.^[^ ^11^ ^]^ The two comparison sets indicate that interfacial charge transfer in Mg‐ion batteries could be the real rate‐determining step rather than solid diffusion. Additionally, such a high energy barrier might also trigger electrolyte decomposition with the formation of solid interphase, which complicates the interfacial transport processes. In light of the findings above, the complexity of cathode electrolyte interfaces has become an emerging challenge for enabling reversible cathode chemistry in RMBs that deserves more attention.^[^ ^9^, ^12^ ^]^

This minireview discusses the previously overlooked interfacial issue at Mg cathodes. To start with, we introduce the most representative interfacial phenomena in different Mg electrolytes and cathode materials to present the current landscape of cathode interfacial chemistry. This is followed by a discussion on the interplay between electrolyte and electrode. On the one hand, we highlight how the solvation structure of Mg^2+^ affects the interfacial mass transport process at the cathode side. On the other hand, recently developed strategies that enhance the cathode‐electrolyte compatibility are discussed, including the progress on cathode‐electrolyte interphase (CEI). Finally, we propose our perspectives on the exploration of practical cathode materials and the functionalized construction of efficient CEI.

## 2 Representative Cathode‐Electrolyte Interfacial Phenomena

**Figure 2:**
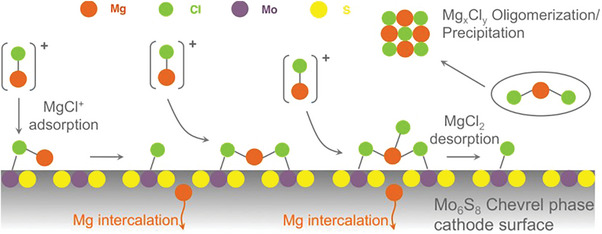
Reaction mechanism diagram of Mg‐ion intercalation into Chevrel phase Mo_6_S_8_ in a chlorine‐based electrolyte. Reproduced with permission.^[^ ^14^ ^]^ Copyright 2015, American Chemical Society.

Looking back to the history and the development of RMBs, there are indeed some cathode materials that show fairly good Mg^2+^ storage capability. Among them, a renowned example is the Chevrel phase Mo_6_S_8_, in which Mg^2+^ can be de‐/inserted reversibly and at a relatively fast charging rate in a so‐called all phenyl complex (APC) electrolyte. Inspiringly, the strong Mg─Cl bonds of electrochemically active Mg*_x_* Cl*_y_* species in the APC electrolyte does not seem to hinder Mg^2+^ insertion into the bulk structure of Mo_6_S_8_.^[^ ^13^ ^]^ To find out the reason behind, Wan et al. conducted a detailed study on the interfacial processes and intercalation mechanisms of Mo_6_S_8_ during magnesiation, revealing a catalytic effect of the Mo_6_S_8_ (100) lattice plane that greatly promotes the dissociation of the Mg*_x_* Cl*_y_* species (**Figure** **2**). The surface‐exposed Mo atoms have strong affinity to Cl, which drastically drives down the dissociation energy from 3.0 to 0.2 eV.^[^ ^14^ ^]^

**Figure 3:**
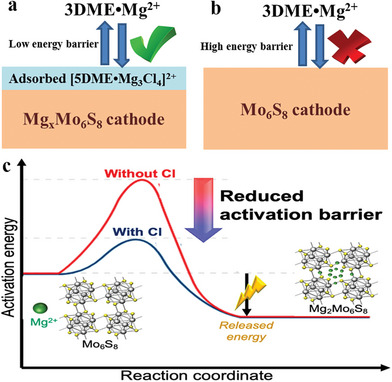
Schematic illustration of the interfacial charge transfer process at Mo_6_S_8_ cathode: a) with or b) without the chloride‐containing species, and c) the corresponding energy profile. Reproduced with permission.^[^ ^16^ ^]^ Copyright 2020, American Chemical Society.

In addition to the catalytic effect of Mo, chlorine might be beneficial for the delivery of Mg^2+^ from the electrolyte bulk to the electrode surface. This is due to the fact that anions are generally solvophobicity, which drives a large population of anion‐containing species to the interface.^[^ ^15^ ^]^ According to a recent study by Aurbach et al., the Mg*_x_* Cl*_y_* species tend to form an adsorption layer on the surface of the Mo_6_S_8_ electrode, which reduced the activation energy for Mg^2+^ transport across the solid‐liquid interface,^[^ ^16^ ^]^ as shown in **Figure** **3**. The adsorption layer can even be well‐maintained after transferring the pretreated Mo_6_S_8_ into a Cl‐free electrolyte solution with the same solvent, which indicates a good stability of the Mg‐Cl clusters. However, the adsorbate rapidly collapses once immersed in other solvents (e.g., acetonitrile) that do not support the formation of similar Cl‐based complexes. These findings highlight the critical role of surface species on the cathode and their stability in governing the energy profile of the interfacial charge transfer processes.

The abovementioned phenomena are based on a two‐phase consideration, where the charge transfer at the phase boundary (interface) of the electrode and electrolyte is essential.^[^ ^17^ ^]^ However, a third phase (interphase) is readily formed in a real battery due to parasitic reactions between the cathode and electrolyte. In this case, the structure, composition, and transport properties of the interphase are critical for the reaction pathway and kinetics of the interfacial processes.

**Figure 4:**
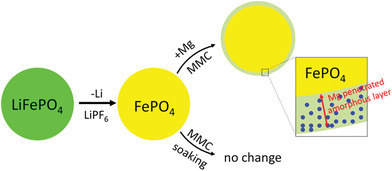
Schematic depiction of the formation process of an amorphous layer on the cathode surfaces of FePO_4_. Reproduced with permission.^[^ ^19^ ^]^ Copyright 2016, American Chemical Society.

In RMBs, the CEI may be derived from the cathode materials. This is due to the sluggish diffusivity of Mg^2+^, which limits its penetration depth into the cathode bulk. The relatively high concentration of Mg^2+^ in the surface region could trigger an irreversible change of the crystal structure with the formation of such an interphase. E.g., Mg^2+^ intercalation into α‐MnO_2_ cathode would lead to the degradation of active particle surface via a conversion reaction mechanism.^[^ ^18^ ^]^ A similar observation was made on olivine FePO_4_, where Mg^2+^ insertion causes strong structural distortion and, hence, surface amorphization, as shown in **Figure** **4**.^[^ ^19^ ^]^ In both cases, the amorphous layer with even a few nanometers thick could already block Mg^2+^ transport toward the particle core and, thereby, have a rather limited capacity. The issue of surface amorphization arises from the thermodynamic instability of the deeply magnesiated phase, which is further constrained by kinetics. However, recent researches have indicated that amorphous cathodes may enhance the diffusion coefficient of Mg^2+^ in solid hosts, thereby facilitating Mg^2+^‐storage in some cathodes.^[^ ^20^, ^21^, ^22^ ^]^ This strategy is feasible only if there is purely kinetic issue. The precise mechanism underlying this amorphization process remains to be elucidated.

**Figure 5:**
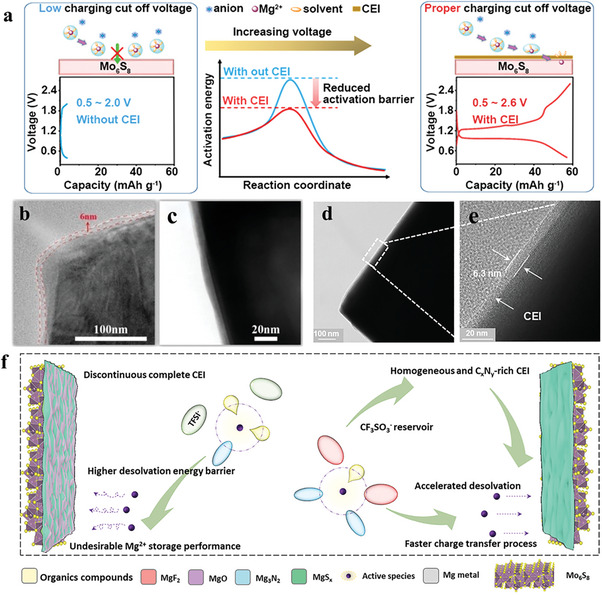
a) Schematic diagram illustrating the function and b,c) typical TEM images of the CEI produced by Mg[B(hfip)_4_]_2_‐based electrolyte on the appropriate charging cut‐off voltage. Reproduced with permission.^[^ ^24^ ^]^ Copyright 2023, Wiley‐VCH. d,e) Typical TEM images and f) schematic of CEI morphologies, composition, and corresponding Mg^2+^ interface transportation behavior on Mo_6_S_8_ cathode in Mg(CF_3_SO_3_)_2_‐based electrolyte with MOEA cosolvent. Reproduced with permission.^[^ ^25^ ^]^ Copyright 2023, Wiley‐VCH.

As a lesson learned from other battery chemistries, the CEI is more commonly formed by electrolyte decomposition at the cathode surface. However, the strategy was previously considered infeasible for the Mg system due to the generally low diffusivity of Mg^2+^.^[^ ^23^ ^]^ Recently, our group reported for the first time the formation of CEI near 6 nm on Mo_6_S_8_ cathode observed by TEM, which enabled reversible Mg^2+^ insertion from a chloride‐free magnesium tetrakis(hexafluoroisopropyloxy)borate (Mg[B(hfip)_4_]_2_) electrolyte(**Figure** **5**b,c).^[^ ^24^ ^]^ By applying a proper charge–discharge protocol, we managed to control the oxidation of [B(hfip)_4_]^−^ anion to a certain degree, yielding a B*_x_* O*_y_*‐contained CEI. It was identified that the B*_x_* O*_y_* species could effectively promote the de‐solvation of Mg^2+^, thereby accelerating the interfacial Mg^2+^ transport kinetics and improving the Mg^2+^‐storage capacity, as depicted in Figure 5a.^[^ ^24^ ^]^ In the same direction, Nuli et al. discovered that the introduction of 2‐methoxyethylamine (MOEA) as co‐solvent in Mg(CF_3_SO_3_)_2_‐based electrolyte is also beneficial for forming a Mg^2+^‐conductive CEI on the Mo_6_S_8_ cathode. Decomposition of the electrolyte produced a homogeneous Mg_3_N_2_ and C*_x_* N*_y_*‐rich layer, which enabled faster charge transfer processes at the cathode interface (Figure 5d–f).^[^ ^25^ ^]^ These two works strongly encourage the design of suitable CEI for RMBs, especially for high‐voltage insertion cathodes. It is worth emphasizing that some reported coating layers on the surface of Mg^2+^‐storage cathodes have the similar effect to artificial CEI. Honma et al. successfully coated Mg–Fe binary oxides onto nano‐sized MgMn_2_O_4_, which significantly inhibited the decomposition of the electrolyte and enhanced the cycling stability.^[^ ^26^ ^]^ Noked et al. formed VS_2_ coatings by reduction on the surface of monodisperse spherical V_2_O_5_ particles, which inhibited electrolyte decomposition and vanadium dissolution and improved intercalation/decalcination kinetics.^[^ ^27^ ^]^ Zhao et al. effectively expedited the kinetics of surface‐redox pseudocapacitance‐dominated charge storage by utilizing an ultra‐thin carbon coating and oxygen vacancies‐enhanced TiO_2_ cathode, demonstrating excellent rate performance.^[^ ^28^ ^]^ Pan et al. catalyzed the de‐solvation by modifying V_2_O_5_ with molybdenum disulfide quantum dots at the cathode electrolyte interface for high‐performance magnesium ion batteries.^[^ ^29^ ^]^ Consequently, customizing the artificial CEI construction can significantly enhance the Mg^2+^‐storage performance of cathode materials.

## 3 Desolvation Process at Cathode Interface

**Figure 6:**
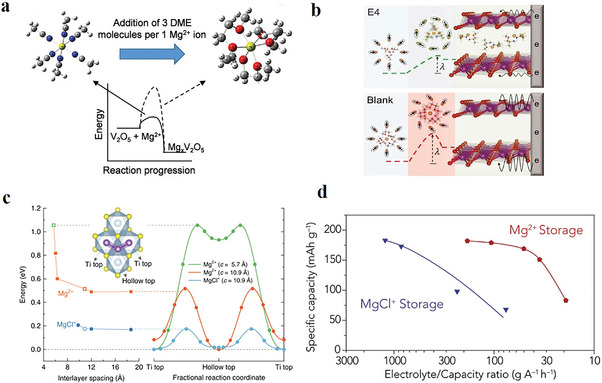
a) Change of the solvation structure in the Mg(ClO_4_)_2_/ACN electrolyte with the addition of DME. Reproduced with permission.^[^ ^30^ ^]^ Copyright 2018, Wiley‐VCH. b) The scheme of concerted ion and electron transfer in the Mg*_x_* MnO_2_ cathode host. Reproduced with permission.^[^ ^33^ ^]^ Copyright 2021, The American Association for the Advancement of Science.  c) Energy profiles for Mg^2+^ and MgCl^+^ diffusion in TiS_2_ with different interlayer spacing. Reproduced with permission.^[^ ^11^ ^]^ Copyright 2017, Springer Nature. d) Performance comparison of Mg^2+^ and MgCl^+^ storage under lean electrolyte conditions. Reproduced with permission.^[^ ^40^ ^]^ Copyright 2019, Elsevier.

Mg^2+^ is subject to a desolvation process before entering the cathode hosts. Due to the strong polarizing effect, the de‐solvation of Mg^2+^ is an energy‐intensive process that can greatly affect the charge transfer mechanism at the cathode electrolyte interface. The influence of solvent on the intercalation of Mg^2+^ was initially examined by Aurbach et al., using a thin‐film V_2_O_5_ electrode in a Mg(ClO_4_)_2_‐based electrolyte. It was found that acetonitrile (ACN) possesses a lower solvation energy than the conventionally used solvent, i.e., dimethoxyethane (DME), which enables reversible Mg^2+^ intercalation into the oxide cathode (**Figure** **6**a).^[^ ^30^ ^]^ However, the presence of even tiny amounts of DME in the Mg(ClO_4_)_2_/ACN electrolyte could already alter the solvation structure, leading to the formation of more stable Mg(DME)_3_^2+^ complexes with a significantly higher energy barrier for the charge transfer across the cathode interface. This result well‐explains the seemingly divergent findings published previously, where some cathode materials exhibited promising electrochemical performance in ACN‐based electrolytes but suffered from rather poor kinetics when testing in full‐cells applying ether‐based electrolytes.^[^ ^31^ ^]^ Note that ACN is not an ideal solvent for RMBs as it passivates Mg metal anode.

To develop a facile desolvation process, searching for a solvent that shows desired solvation strength with Mg^2+^ and high compatibility with Mg metal anode is imperative. “Desired” is termed aiming at a balance between Mg^2+^ desolvation and dissociation of the electrolyte compound. However, finding such a solvent is still challenging as ethers (such as tetrahydrofuran and glyme) are almost the only solvent type to date that allows reversible Mg plating/stripping. Drews et al. studied the de‐solvation of Mg^2+^ in Mg[B(hfip)_4_]_2_‐based electrolytes and investigated the influence of different glymes on the battery performance. It was found that the initial step to release the first coordination site is the most energy‐consuming process, where diglyme (G2) and tetrahydrofuran (THF) show a lower value among others.^[^ ^32^ ^]^ Although focusing on the anode process, the kinetic model developed in this study could also be extended for the de‐solvation process at the cathode. The solvation structures in various Mg electrolytes have been summarized in **Table** **1**.

**Table 1:** Summary of the solvation structures in various Mg electrolytes.

Mg salt	Solvent	Solvation structure	References
Mg(ClO_4_)_2_	ACN	Mg^2+^(6ACN)	[30]
Mg(ClO_4_)_2_	ACN:DME	Mg^2+^(3DME)	[30]
Mg(ClO_4_)_2_	ACN	/	[31]
Mg[B(hfip)_4_]_2_	G1	Mg^2+^(3G1)	[32]
Mg[B(hfip)_4_]_2_	G2	Mg^2+^(2G2)	[32]
Mg[B(hfip)_4_]_2_	G3	Mg^2+^(2G3)	[32]
Mg[B(hfip)_4_]_2_	G4	Mg^2+^(2G4)	[32]
Mg[B(hfip)_4_]_2_	THF	Mg^2+^(6THF)	[32]

As completely replacing the ether solvent is challenging, introducing cosolvent or additive is another effective strategy for modifying the solvation structure of Mg^2+^. Wang et al. reported a family of methoxyethylamine chelants as cosolvent for Mg(TFSI)_2_/DME electrolytes, which greatly promotes interfacial charge transfer at both cathode and anode.^[^ ^33^ ^]^ It was found that the methoxyethyl‐amine could replace DME in the solvation sheath by offering stronger chelating site. However, the heterogeneous coordination (by oxygen in the ether group and nitrogen in the amine group) renders a more flexible solvation structure with a lower de‐solvation barrier than the DME‐solvated structure. With the novel electrolyte design, the authors demonstrated a RMB with an impressively high energy density of 412 Wh kg^−1^ using a Mg_0.15_MnO_2_ cathode (Figure 6b). In the same direction, Nuli et al. employed nitrogenous 2‐methoxyethylamine (MOEA) as a co‐solvent to regulate the coordination behavior of cations/anions in Mg(CF_3_SO_3_)_2_/G2 electrolyte. The addition of MOEA could, on the one hand, soften the solvation structure of Mg^2+^. More importantly, the decomposition products of MOEA‐tailored cationic/anionic complexes protect Mg metal anode and Mo_6_S_8_ cathode with Mg_3_N_2_ and C*_x_* N*_y_*‐rich layers, respectively, which not only suppress interfacial side reaction but also accelerate Mg^2+^ transport.^[^ ^25^ ^]^ The beneficial effect of MOEA‐derived interphase in the Mg(CF_3_SO_3_)_2_/G2 electrolyte was also reported by Pan et al.^[^ ^34^ ^]^ In addition to solvents, electrolyte additives may modulate the solvation structure by introducing strong chelating auxiliary anions. Fine‐tuning can be made by balancing the relative coordination strength between the main anions and the auxiliary anions with respect to Mg^2+^, which generates synergetic effects to accelerate cathode reactions.^[^ ^35^ ^]^

On the other hand, cointercalation chemistries were explored, targeting circumventing the sluggish desolvation process at the cathode electrolyte interface. Cointercalation chemistry typically involves an auxiliary agent, either solvent or anion, that intercalates into the cathode in the form of solvated Mg^2+^ clusters. In a broad range of Mg electrolytes, the addition of Cl^−^ significantly improves their compatibility and reversibility at the Mg metal anode with the formation of electrochemically active Mg*_x_* Cl*_y_* ^+^ species.^[^ ^36^, ^37^, ^38^ ^]^ Yao et al. proposed to intercalate MgCl^+^ instead of bare Mg^2+^ into the cathode host, hoping to bypass the dissociation of Mg─Cl, which possesses a high energy barrier >3 eV.^[^ ^14^ ^]^ Due to its monovalency and large size, MgCl^+^ also has a lower charge density, therefore rendering accelerated solid diffusion kinetics in cathode hosts.^[^ ^39^ ^]^ As reported, the substitution of Mg^2+^ by intercalated MgCl^+^ in an interlayer‐expanded TiS_2_ (*d* = 10.9 Å) led to a drastic decrease in the diffusion barrier from 0.51 to 0.18 eV (Figure 6c), which corresponds to more than five orders of magnitude higher in diffusivity.^[^ ^11^ ^]^ With these synergetic effects, the pillared TiS_2_ provided a capacity of up to 400 mAh g^−1^ and stable cycling of up to 500 cycles. Nevertheless, the corrosive nature of Cl^−^ raised concerns about the compatibility issue with high‐voltage oxide cathodes.^[^ ^23^ ^]^ Additionally, MgCl^+^ storage results in a low specific energy at the cell level. Especially under lean electrolyte conditions, the specific energy of systems with MgCl^+^ storage might be reduced by 2/3 compared to the Mg^2+^ storage, as depicted in Figure 6d.^[^ ^40^ ^]^ Similar challenges are encountered in other co‐intercalation processes involving solvated Mg^2+^ with solvent molecules.^[^ ^33^, ^41^ ^]^ The cointercalated Mg^2+^‐containing species in those reported works have been summarized in **Table** **2**.

**Table 2:** Summary of the co‐intercalated species in various Mg electrolytes.

Mg salt	Solvent	Co‐intercalated species	References
Mg(TFSI)_2_	M1:DME	Mg^2+^(M1)	[33]
Mg(CF_3_SO_3_)_2_	MOEA:G2	/	[25]
Mg(CF_3_SO_3_)_2_	MOEA:DME:G2	Mg^2+^(3MOEA)	[34]
Different anions	G2	Mg^2+^(G2)(2Cl^−^)_2_	[35]
Chlorides	/	Mg*_x_* Cl*_y_* ^+^ species	[11, 14, 23, 36, 37, 38]

## 4 The Compatibility between Cathode and Electrolyte

**Figure 7:**
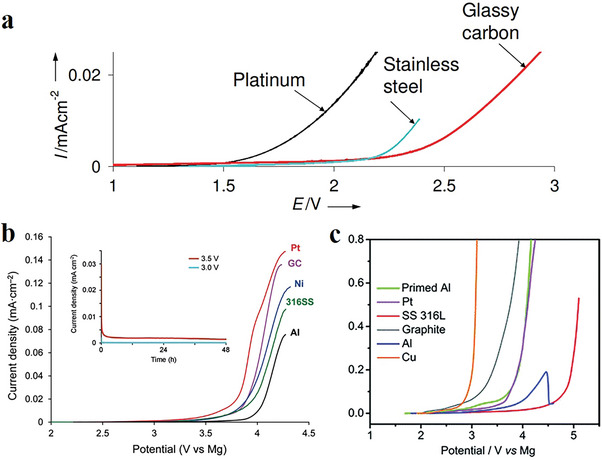
Linear sweep voltammograms of a) Mg(BH_4_)_2_/THF. Reproduced with permission.^[^ ^45^ ^]^ Copyright 2012, Wiley‐VCH. b) Mg(CB_11_H_12_)_2_/G4 (inset: chronoamperometry on 316SS disk electrodes (area = 1.33 cm^2^)). Reproduced with permission.^[^ ^47^ ^]^ Copyright 2015, Wiley‐VCH. and c) Mg[B(hfip)_4_]_2_/DME measured on various electrodes at a scan rate of 5 mV s^−1^. Reproduced with permission.^[^ ^46^ ^]^ Copyright 2017, Royal Society of Chemistry.

In search of a suitable electrolyte for RMBs, initial attempts were made to improve the anodeelectrolyte compatibility, which resulted in the development of organometallic compounds as the first‐generation Mg electrolyte.^[^ ^42^ ^]^ To enhance the anodic compatibility of these electrolytes, Aurbach et al. introduced a strong Lewis acid AlCl_3_ into phenyl magnesium chloride (PhMgCl), forming the abovementioned APC electrolyte.^[^ ^13^ ^]^ By stabilizing the Mg─C bonds of the Grignard compounds, the APC electrolyte provides a larger electrochemical window up to 3 V, which enables the screening of cathode materials with moderate operating voltage. Nevertheless, the addition of chloride into electrolyte leads to the formation of Cl‐based Lewis acidic moieties, which are corrosive to oxide cathodes and conventionally used aluminum current collectors.^[^ ^43^ ^]^ Moreover, their nucleophilic character further constrains their chemical compatibility with electrophilic electrodes that contain S─S bonds or redox active organics.^[^ ^44^ ^]^ In these regards, non‐nucleophilic Mg electrolytes without chloride components are desired. As a lesson learned from hydrogen storage, Mohtadi et al. reported Mg(BH_4_)_2_ as the first compound of this class, but with limited anodic stability of ≈2 V versus Mg (**Figure** **7**a).^[^ ^45^ ^]^ Despite that, this work inspired further design of Mg compounds with B‐centered fluorinated alkoxyborates, or Al‐centered fluorinated alkoxyaluminates,^[^ ^46^ ^]^ or carborane clusters.^[^ ^47^, ^48^ ^]^ All these anions are stable against electrochemical oxidation up to 4 V versus Mg (Figure 7b,c) but are also chemically inert to aggressive cathodes, including elemental S and redox‐active compounds. In addition, they have rather small association strength, which gives rise to a high degree of dissociation of their Mg salts in ether solvent, therefore guaranteeing sufficient Mg^2+^ transport. Due to their superior chemical stability and electrochemical compatibility, simple‐salt Mg compounds with weakly coordinating anions present a new research direction toward more practical Mg electrolytes, which can serve as a new benchmark for cathode screening.

In addition to anions, the selection of solvent is another pivotal factor in determining cathode‐electrolyte compatibility. Alkylcarbonates are the prevalent choice of Li^+^‐conducting electrolytes in commercial Li‐ion batteries but are passivating to Mg. Currently, Mg^2+^‐conducting electrolytes that exhibit highly reversible Mg plating/stripping behavior commonly use ether solvents. However, the oxidative stability of ether solvent is limited to approximately 3.5 V versus Mg.^[^ ^23^ ^]^ The electrochemical window of ether‐based electrolytes can be slightly expanded by forming a solvated Mg^2+^ structure. Mandai et al. proposed a [Mg(G4)][TFSI]_2_/ionic liquid electrolyte (G4 = tetraglyme) in which all G4 molecules are coordinated to Mg^2+^.^[^ ^49^ ^]^ By fixing the free solvent, the oxidation stability of the electrolyte was enlarged by 0.7 V. Nevertheless, solvent decomposition at high voltages and extended cycles was still present. In addition, the cross‐talk between the cathode and anode might further exacerbate the electrolyte decomposition. This is still an ongoing challenge that awaits more careful design of electrolytes. Inspiringly, the recent discovery of viable cosolvents, either methoxyethyl‐amine^[^ ^33^ ^]^ or triethyl phosphate,^[^ ^50^ ^]^ has demonstrated its feasibility to enable stable cycling for up to 3.5 V and up to 400 cycles.

**Figure 8:**
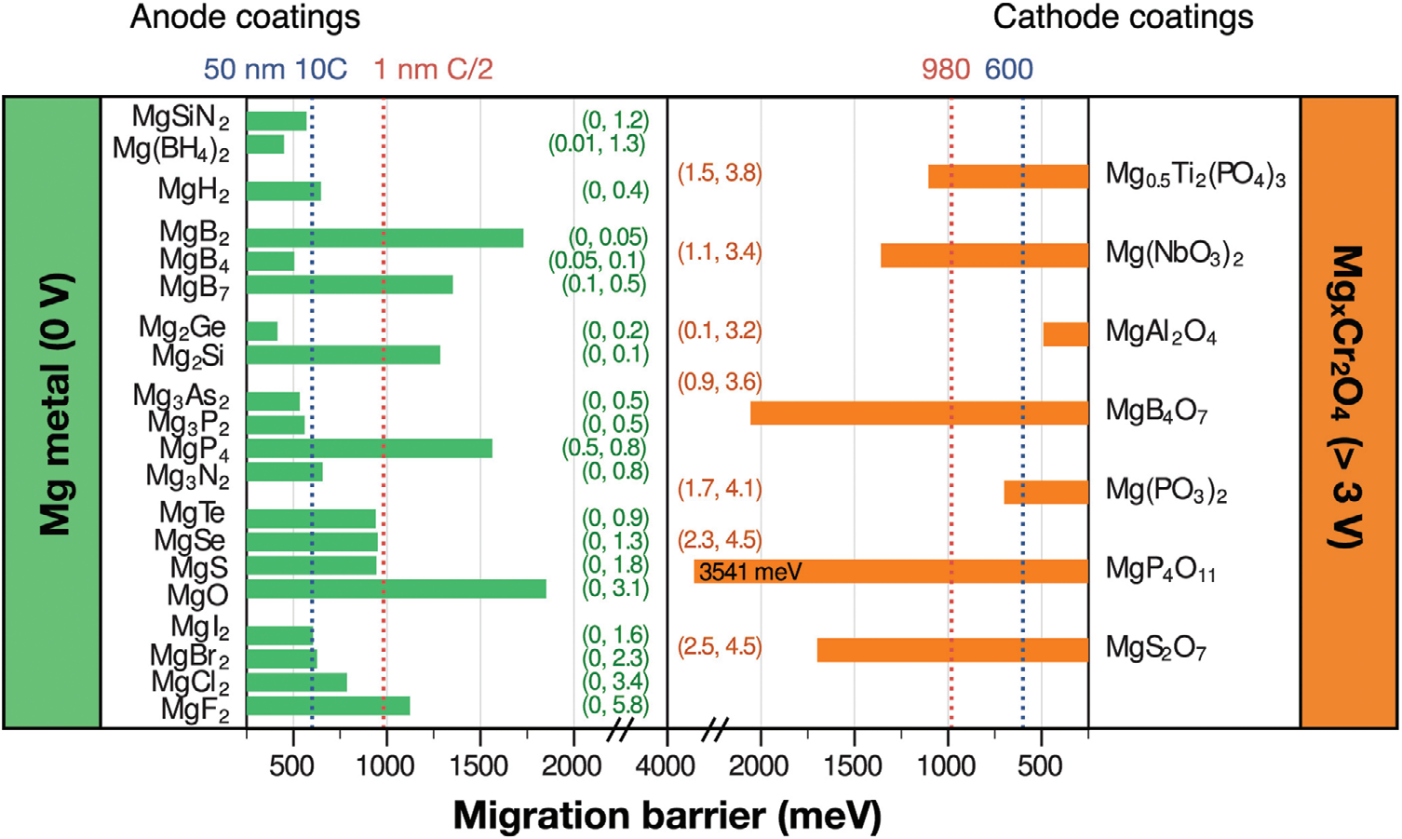
Calculated migration barriers for anode (left) and cathode (right) coating candidates. Dotted vertical lines indicate strict threshold (≈600 meV, blue) and lenient threshold (≈980 meV, red) *E*_m_^max^ values. The numbers in brackets provide the (reductive, oxidative) stability limits in V versus Mg metal. Reproduced with permission.^[^ ^52^ ^]^ Copyright 2019, American Chemical Society.

Constructing Mg^2+^‐conducting electron‐insulating interphase on a cathode is another viable approach to kinetically widen the electrochemical window of Mg electrolytes. The CEI can be formed in situ during electrochemical cycling, as mentioned above. Recent studies on Mo_6_S_8_ cathode have identified B*_x_* O*_y_* species derived from Mg[B(hfip)_4_]_2_/DME electrolyte and Mg_3_N_2_ and C*_x_* N*_y_* species from Mg(CF_3_SO_3_)_2_/G2‐MOEA electrolyte, respectively, as viable CEI components.^[^ ^25^ ^]^ The feasibility of forming CEI on high‐voltage cathode materials from those electrolytes deserves further validation, which may open up an avenue toward high‐voltage RMBs. Alternatively, an artificial CEI layer could be directly coated on the cathode material prior to cell assembly. Canepa et al. conducted a high‐throughput computational screening of a broad range of Mg‐containing compounds, applying ion mobility, electronic band gaps, and stability requirements as descriptors. As predicted, Mg(PO_3_)_2_, and MgP_4_O_11_ are potential candidates against high‐voltage cathode materials up to ≈3.0 V, as illustrated in **Figure** **8**.^[^ ^51^, ^52^ ^]^ In addition to material consideration, the structure of CEI also affects its functionalities. A porous MgF_2_ (activation energy of > 1000 meV) coating with ≈1 nm thick could also conduct Mg^2+^ effectively.^[^ ^53^ ^]^ The result highlights the importance of constructing structural diffusion pathways within the artificial CEI layer, e.g., by regulating the coating thickness, creating a porous coating layer, or introducing vacancies, etc.

## 5 Perspectives and Challenges

Interfacial chemistry in batteries is highly dependent on the electrolyte formulation. The bivalency and high charge density of Mg^2+^ requires unconventional electrolyte design with respect to the Li counterpart, targeting sufficient ion mobility. This leads to a strong solvation structure of Mg^2+^ but also a more prominent ion pairing effect. The former would generate a high activation energy barrier for interfacial Mg^2+^ transport, while the latter normally reduces the oxidative stability of electrolyte anions. In addition, the intrinsic sluggish mobility of Mg^2+^ in solid makes it challenging for the design of any solid interphase between cathode and electrolyte. As a result, interfacial chemistry in Mg batteries is more complicated than in Li systems and requires a more fundamental understanding of interfacial phenomena and transport mechanisms. Based on the literature review and our experience with the topic, we provide our perspectives as follows.

- There is complex reaction equilibrium between various Mg*_x_* Cl*_y_* ^−^ species in the Cl‐based electrolyte. Whereas, simple‐salt Cl‐free electrolytes offer a more stable solution environment with only solvated Mg^2+^ and electrolyte anions. Accordingly, the simple‐salt Cl‐free electrolyte could be a better benchmark system for the modification of solvation structure and a better model system for a fundamental understanding of the de‐solvation mechanism.
- A straightforward strategy to soften the solvation structure of Mg^2+^ is to search for solvents with weaker coordination strength. However, this has to compromise the solubility and ionic conductivity of the electrolyte. In this regard, the development of anions with even weaker coordinating strength than the state‐of‐the‐art, e.g., [B(hfip)_4_]^−[^ ^54^ ^]^or [Al(hfip)_4_]^−[^ ^55^ ^]^ would be beneficial, which provide a larger room for the selection of solvent.
- The other way around, recent studies^[^ ^33^ ^]^ demonstrated the feasibility of employing stronger chelants as co‐solvents to modify the solvation structure of Mg^2+^, which even facilitated the interfacial charge transfer by enabling easier de‐solvation. Designing solvents with heterogeneous coordination sites and flexible molecular structures emerges as a new research direction.
- The strategy to construct a heterogeneous coordination structure of Mg^2+^ is actually in line with another finding, which identified that the initial de‐solvation process to free the first coordination site is the most energy‐intensive process.^[^ ^32^ ^]^ Therefore, further efforts on Mg^2+^ de‐solvation would contribute to clarifying the rate‐limiting steps so that improvement strategies can be better developed. Furthermore, the complicated reaction mechanism for Mg^2+^ desolvation calls for more dedicated investigation and delicate structural design of both the inner shell and outer shell of the solvation structure.^[^ ^50^ ^]^
- While cathode interfacial chemistries in RMBs remain largely unexplored, the interfacial process at the Mg anode site has been intensively studied. The established principle and mechanism for Mg^2+^ de‐solvation at the anode site offer valuable insights for a similar cathode process. However, attention should be paid to the difference between the de‐solvation process at the cathode and at the anode. One major difference is that the anode de‐solvation is accompanied by the reduction of Mg^2+^, where charge transfer to the cation core through the solvates might have a significant impact on the thermodynamics and kinetics of the process.
- Because of the limited penetration depth of Mg^2+^, the size effect is outstanding in most cathode materials, even those sulfide cathodes with moderate voltage.^[^ ^56^ ^]^ To fully exploit the electrochemical performance and achieve a more homogeneous magnesiation state, reducing the particle size is a practical approach.^[^ ^57^ ^]^ While these strategies enable improved evaluation of the Mg hosts, attention should be paid to more severe side reactions resulting from larger surface areas. Nanoparticles typically exhibit increased (electro‐)chemical reactivities, including other supposedly inert cell components.^[^ ^58^ ^]^ Since side reactions affect the measured capacity, carefully evaluating these materials and their possible interphases is required.
- Building a functional CEI is a promising strategy, especially via in situ formation during electrochemical cycling by a controlled decomposition of electrolytes. Recent studies have demonstrated the feasibility of constructing electrolyte‐derived CEI on Mo_6_S_8_. More efforts in this direction could be made to establish the correlation between solvation structure and the decomposition products. Based on that, the ultimate expectation is to transfer the concept to a high‐voltage cathode.
- Artificial CEI also deserves further investigation. This allows high throughput screening to identify functional CEI components, which could also feed back to the electrolyte design for building suitable in situ CEIs. At the material level, searching from a wide chemical space other than Mg‐containing compounds may yield more potential candidates. In addition, materials that already show fair Mg conductivity could be employed as a coating layer for the cathode, such as Mo_6_S_8_, transition metal sulfides, or even polymers. A successful development of an artificial CEI may extend the application of Cl‐based electrolytes towards oxide cathodes by avoiding corrosion issues and/or taking advantage of the surface catalytic effect for easier dissociation.

Overall, cathode‐electrolyte interfacial chemistry is essential for the development of RMBs, especially at the current stage, where remarkable progress has been made in electrolyte and Mg anode, which leaves the exploration of viable cathode as a bottleneck issue. As Mg^2+^ suffers from a high energy barrier across the cathode interface, a delicate design of the interfacial process would pave the way for better screening of cathode materials. This requires mutual efforts from electrolyte development and interfacial engineering for a comprehensive understanding of the interfacial phenomena and charge transfer mechanisms. The ultimate goal is to construct a thermodynamically stable interface and to establish kinetically favorable interfacial reactions towards compatible cathode‐electrolyte interfacial chemistry, by which to push the boundary of what can be achieved by RMBs.

## Conflict of Interest

The authors declare no conflict of interest.
